# The effectiveness of school-based physical activity interventions on health-related fitness and BMI in South Korea: a three-level meta-analysis

**DOI:** 10.3389/fped.2026.1849629

**Published:** 2026-06-03

**Authors:** Jihyun Song, Wonseok Choi

**Affiliations:** Department of Kinesiology, Keimyung University, Daegu, Republic of Korea

**Keywords:** adolescents, children, context specificity, physical activity program, health-related fitness, school-based exercise, BMI

## Abstract

**Background:**

As empirical evidence has accumulated, systematic reviews and meta-analyses have documented the effectiveness of school-based physical activity interventions on health-related fitness and BMI. Nevertheless, the majority of reviews have been conducted across different contexts, leaving a lack of evidence on the effectiveness in unique contexts, such as South Korea. The purpose of this three-level meta-analysis was to determine the effectiveness of school-based physical activity interventions on health-related fitness and body mass index (BMI) in South Korea.

**Methods:**

Adhering to Preferred Reporting Items for Systematic Reviews and Meta-Analysis (PRISMA) guidelines, this study conducted a literature search, selected eligible intervention studies, and collected relevant data. Then, a three-level meta-analysis was performed in R software with moderator analysis, heterogeneity testing, and publication bias assessment.

**Results:**

The three-level meta-analysis of the included studies (*n* = 24) demonstrated a small-to-moderate overall effect (Hedges' *g* = 0.33, *p* < 0.001). Heterogeneity was found at Level 2 with $\tau^{2}$ of 0.08 ($I^{2}$ = 46.28%), which was statistically significant (LRT=25.52, *p* < 0.001). This within-study variance was evident in the outcome-specific effect sizes, such as muscular endurance (Hedges' *g* = 0.48, *p* < 0.001), muscular strength (Hedges' *g* = 0.46, *p* < 0.001), flexibility (Hedges' *g* = 0.23, *p* < 0.001), BMI (Hedges' *g* = 0.28, *p* = 0.01), and body fat % (Hedges' *g* = 0.24, *p* = 0.04), but not in cardiorespiratory endurance (*p* > 0.05). Weekly duration (min/week) emerged as a sole significant moderator (*QM*_(*df*_ *_=_* _2)_ = 5.90, *p* = 0.04). Specifically, the 100–150 min/week dose demonstrated a significant effect size (*β* = 0.28, *SE* = 0.12, *p* = 0.02), compared with <100 min/week and >150 min/week (*p* > 0.05).

**Conclusion:**

Consistent with previous research, the findings of this study suggest that school-based physical activity interventions may improve health-related fitness and BMI in Korean children and adolescents, providing potential empirical evidence to inform future program development. However, generalizability of the findings should be limited due to methodological concerns, such as small sample sizes across intervention studies and the quality of the intervention studies.

## 1 Introduction

Pediatric obesity is emerging as a critical global health priority in the 21st century (1). According to the World Health Organization (WHO), over 390 million children and adolescents aged 5–19 were overweight or obese in 2022 (2). The prevalence of overweight and obesity in this age group has escalated significantly, rising from 8% in 1990 to 20% in 2022. The primary concern about this prevalence is that pediatric obesity is often associated with non-communicable diseases (NCDs), such as hypertension, type 2 diabetes, and cardiovascular disease (2, 3), and adverse psychosocial effects, such as school performance, quality of life, anxiety, depression, and stigma (2, 4). In addition, pediatric obesity is likely to affect overweight and obesity in adulthood, thereby increasing the risk of such NCDs compared with those with a healthy weight.

One of the fundamental determinants of pediatric obesity is an energy imbalance between intake and expenditure, a process of complex interactions among biological, psychological, social, and environmental factors (2, 5). Previous research has revealed that weight-loss interventions aimed at energy balance in children and adolescents, such as dietary control, physical activity (PA), and behavioral therapy, in community and school settings have positive effects on decrease in body mass index (BMI), body weight, and body fat %, while improving metabolic function, such as insulin sensitivity and glycemic control (6, 7). In particular, PA is a relatively modifiable factor of interventions that not only facilitates weight loss but also health-related fitness, which can mitigate the metabolic risks associated with pediatric obesity (8).

To obtain the full benefits of health-related fitness and BMI, WHO (8) recommends at least 60 min of daily moderate-to-vigorous intensity physical activity (MVPA) for children and adolescents. In addition, it was advised to engage in muscle- and bone-strengthening activities at least three times per week. However, physical inactivity and declines in health-related fitness are prevalent across countries. Using data from 146 countries, including 1.6 million children and adolescents aged 11–17 years, Guthold et al. (9) revealed that approximately 81.1% did not meet the WHO guidelines. For health-related fitness, Tomkinson et al. (10), analyzing data from 137 studies, with 965,264 children and adolescents from 19 countries between 1981 and 2014, revealed that cardiorespiratory fitness declined by 7.3% over 33 years. Similar declining trends have also been observed in muscular endurance, muscular strength, and flexibility (11, 12). This alarming level of physical inactivity and the decline in health-related fitness highlights the need to develop effective PA interventions.

Schools have been recognized as one of the best venues to conduct PA interventions through before- and after-school PA programs, sport/PA clubs, PA breaks, and physical education classes (13). Research has provided extensive empirical evidence on the effects of school-based PA interventions for health-related fitness and BMI. For example, Eather et al. (14) implemented an eight-week, high-intensity, in- and out-of-school PA program in Australia that included health and physical education (60 min per week), daily PA breaks during recess and lunch, and home activities (three 20-minute sessions per week). The study revealed that the program contributed to fostering elementary school students' cardiorespiratory fitness and flexibility while decreasing their BMI at the 6-month follow-up. Similarly, Lee (15) demonstrated that the students in a 12-week school-based Kinball sports club program improved cardiorespiratory fitness, and muscular endurance, with a decrease in body fat %, compared with those in the control group. With numerous school-based PA interventions implemented globally, recent studies have synthesized the empirical evidence (16–19). For instance, addressing the role of PA intensity, Zhou et al. (19) compared high-intensity PA interventions (e.g., HIIT, resistance training) with low-to-moderate intensity PA interventions. This study reported that high-intensity PA was superior for improving cardiopulmonary function, muscular endurance, and muscular strength and for decreasing BMI, waist circumference, and body fat. The benefits of HIIP programs at school were repeatedly reported in other systematic reviews (20). Collectively, these review studies suggest that school-based PA interventions, such as HIIT programs, may contribute to improvements in health-related fitness and a decline in BMI.

Despite the empirical evidence, the effectiveness of school-based PA interventions remains unclear for three reasons. First, most reviews have focused primarily on HIIT [e.g. (20, 21),] or different intensities (19), limiting an in-depth understanding of the effects of diverse school-based PA programs in terms of gender, intervention types, and approach to PA interventions (e.g., public health, education, recreation). Second, existing meta-analyses adopted traditional meta-analytic approaches that assume independence among multiple effect sizes. However, health-related fitness components and BMI are inherently interrelated, and thus treating them as independent can lead to inflated results or reduced statistical power (22). Lastly, and more importantly, previous reviews have largely been cross-country or trans-contextual. Although research findings across countries and cultures have provided multiple implications for designing generalizable interventions (19–21), they often overlook unique socio-cultural and structural nuances of the very country/region for populations and settings, which may hinder designing optimal PA interventions (23, 24). Thus, simply assuming that the effectiveness of PA interventions on health-related fitness and BMI is consistent across countries and cultures appears to limit the potential of understanding the authentic efficacy of these interventions in varying contexts. For example, South Korea can be a unique context where the effectiveness of school-based PA interventions may significantly differ from other contexts due to structural environmental complexity. In this context, physical inactivity among children and adolescents has continuously been the highest across 146 countries (9). The Korean school system is highly standardized at the national level in terms of curriculum and school operations. Korean children and adolescents often face strong academic expectations and demands, which may limit opportunities to engage in PA and improve health-related fitness (25). In particular, approximately 64.9% of Korean students do not have sufficient time for after-school PA as they, in general, attend private tutoring to enhance academic achievement in core academic subjects such as literature, mathematics, science, and English (25). These unique socio-contextual factors suggest that in the Korean school context, the effectiveness of school-based PA interventions observed across countries and cultures may vary. A meta-analysis in this Korean context would provide context-specific evidence on the effectiveness of school-based PA interventions.

The purpose of this study, therefore, was to determine the effectiveness of school-based PA interventions on health-related fitness and BMI in South Korea, adopting a three-level meta-analytic model. It is expected that the findings of this study will provide empirical evidence on context-specific effectiveness of school-based PA interventions, serving as a valuable benchmark for different cultures and contexts facing similar challenges. This study would also enhance methodological rigor through the three-level meta-analytic model that accounts for dependencies among effect sizes within and between studies.

## 2 Methods

Adhering to the Preferred Reporting Items for Systematic Reviews and Meta-Analysis (PRISMA) guidelines (26), this study conducted a meta-analysis to demonstrate the effectiveness of school-based PA interventions on health-related fitness and BMI. The pre-established inclusion and exclusion criteria served as a tool for selecting eligible studies. We searched potential articles using multiple keyword combinations across electronic databases. From the selected studies, data were extracted and used to calculate effect sizes. Then, a three-level meta-analysis was performed.

### 2.1 Inclusion and exclusion criteria

We pre-established inclusion criteria for the literature search as follows: (a) school-based interventions conducted in South Korea (e.g., before- and after-school PA, physical education, club sports, student-organized PA club, PA break), (b) a sample of K-12 students (i.e., elementary, middle, and high schools), (c) participants without physical or health illness, (d) a pre-post-control design, (e) measured health-related fitness outcomes (e.g., muscular strength, muscular endurance, cardiorespiratory endurance, flexibility, BMI, body fat %), (f) reported pre- and post-statistics (e.g., *M, SD*, sample size, pre- and post-measured *r*, if available), and (g) peer-reviewed studies written in English or Korean. Intervention studies that did not meet the criteria were excluded, such as non-school-based intervention studies (e.g., correlational, qualitative studies), non-pre-post-control designs, interventions that did not report pre- and post-statistics, studies involving university students, and grey literature (e.g., thesis, dissertation, conference research papers).

### 2.2 Literature search strategies

An electronic literature search was performed in (a) Korean databases, including Korea Citation Index, DBpia, Korean Studies Information Service System, Scholar, and eArticle; and (b) international databases, including Academic Search Complete (EBSCO), APA PsycInfo, Education Source, ERIC, SPORTDiscus, Web of Science, and Scopus. This search was conducted using keyword combinations at an abstract level as follows: school physical education OR school physical activity OR school-based physical activity OR school sports OR school sports club OR after-school physical activity OR before-school physical activity OR physical activity break AND intervention OR program OR effect OR change AND health OR health-related fitness OR BMI OR muscular endurance OR muscular strength OR cardiorespiratory endurance OR flexibility OR body composition OR body fat mass OR percentage of body fat OR muscle mass OR percentage of muscle OR lean body mass AND Korea. The initial search in the Korean databases was conducted on September 16, 2025. The literature search was then expanded and updated on April 21, 2026, to include both Korean and international databases without any date restrictions, ensuring that all relevant studies up to that date were included.

### 2.3 Data collection process

Reviewing all studies that met the inclusion criteria, we independently collected data on statistics (e.g., pre-post *M, SD*, sample size) measured outcomes (e.g., BMI, muscular endurance, muscular strength), as well as relevant characteristics of the studies, including school level, gender, intervention type, intervention approach, study design, analyses, intensity, and weekly duration. Table 1 presents a summary of the coding sheet, including 9 codes classified into 39 code categories. As seen in Table 1, several body composition indices were identified in the data collection process; however, those reported in only one study, such as fat mass and lean body mass, were excluded from the analysis of outcome-specific effects to avoid potentially biased estimates, ensuring statistical stability.

**Table 1:** Summary of the coding sheet.

Code categories	Specific codes
Outcome	Cardiorespiratory endurance, muscular endurance, muscular strength, flexibility, BMI, body fat %, fat mass, lean body mass
School level	Elementary, middle, high, combined (elementary & middle)
Gender	Female, female and male, male
Program type	Physical education, sports club, after-school PA, before-school PA, physical activity break, student-organized club, in-and-out of school PA, Combined (physical education & After-school PA)
Approach	Educational, public health, recreational
Study design	Randomized controlled trial, non-randomized design
Intensity	Low-to-moderate, moderate, moderate-to-vigorous, vigorous
Weekly duration	<100 min/week, 100–150 min/week, >150 min/week
Statistical analysis	Dependent *t*-test, independent *t*-test, repeated measures ANOVA, ANCOVA

We shared the coding sheets and data and discussed any discrepancies to ensure inter-rater reliability. For example, there was a discrepancy in categorizing weekly duration. In a research meeting, we discussed and decided to categorize it into <100 min/week, 100–150 min/week, and >150 min/week based on the standard class periods (40–50 min) and the empirical distribution of the included studies. In addition, based on the details and focus of the interventions described in the original studies, we categorized PA interventions; we coded after-school sports club activities as “sports club,” while coding PA programs that were simply operationalized after school (not sports club) as “after-school PA.”

### 2.4 Risk of bias assessment

The quality of the included intervention studies was assessed using two risk-of-bias tools: the revised Risk of Bias in Randomized Trials (RoB 2) (27) and the Risk of Bias in Non-Randomized Studies of Interventions (ROBINS-I) (28). RoB 2 is an assessment tool for randomized intervention studies, including five domains: the randomization process, deviations from intended interventions, missing outcome data, outcome measure, and selection of the reported results. The risk of bias in each domain and the overall bias were coded as “low,” “some concerns,” and “high,” based on the judgments in response to the signaling questions. ROBIN-I for non-randomized intervention studies consists of seven domains: confounding, selection of participants, classification, deviations from intended interventions, missing data, outcome measure, and selection of the reported results. Domain-specific and overall risk of bias were categorized as “low,” “moderate,” “serious,” and “critical” based on responses to the leading questions.

### 2.5 Calculating effect sizes

Effect sizes for each measure outcome were computed using the means, standard deviations, and sample sizes in the original articles. Because of small sample bias, an adjustment was computed, yielding Hedges' *g*. Effect sizes were calculated based on the effect size formula for the pre-post-control design recommended by Morris (29), as follows:

$$\;\mathrm{Hedg}e^{{}^{\prime }}s\;g=(C)\left[\frac{\left(M_{\;post,E}-M_{\;pre,E})-(M_{\;post,C}-M_{\;pre,C}\right)}{SD_{\;pre}}\right],$$

$$SD_{\;pre}\;=\sqrt{\frac{(n_{E}-1)SD_{\;pre,E}^{2}+(n_{C}-1)SD_{\;pre,C}^{2}}{n_{E}+n_{C}-2}},\mathrm{and}$$

$$C\;=1-\frac{3}{4(n_{E}+n_{C}-2)-1},$$

where C is the adjustment for bias correction; $M_{\;post,E}$ is the post-measure mean of the experimental group; $M_{\;pre,E}$ is the pre-measure mean of the experimental group; $M_{\;post,C}$ is the post-measure mean of the control group; and $M_{\;pre,C}$ is the pre-measure mean of the control group. $SD_{\;pre}$ is a pooled standard deviation of the pre-measure standard deviations of the experimental and control groups, where $n_{E}$ is the sample size of the experimental group; $SD_{\;pre,E}^{2}$ is the squared pre-measure standard deviation of the experimental group; $n_{C}$ is the sample size of the control group; and $SD_{\;pre,C}^{2}$ is the squared pre-measure standard deviation of the control group. Because the effect sizes were inconsistent in direction (e.g., muscular strength vs. BMI), the negative direction was reversed when it indicated a beneficial effect, to ensure that the positive direction reflected the intervention's positive effect. The effect size variance was computed using the following formula:

$$V\;=2(C^{2})(1-r)\left(\frac{n_{E}+n_{C}}{n_{E}n_{C}}\right)\left(\frac{n_{T}+n_{C}-2}{n_{C}+n_{C}-4}\right)\left(1+\frac{g^{2}}{2(1-r)\left(\frac{n_{T}+n_{C}}{n_{C}n_{C}}\right)}\right)-g^{2},$$

where *r* is the pooled pre-post-measure correlation of the experimental and control groups used for the variance estimate. However, since most of the *rs* were not reported in the original articles, three correlations (*r* = 0.3, 0.5, 0.7) as commonly used in the literature were applied to conduct a sensitivity analysis for the robustness of the results. An *r* value of 0.5 was adopted in the meta-analysis if the sensitivity analysis demonstrated no significant difference in the overall effect sizes, as recommended in the literature (30).

### 2.6 Analysis

Multiple effect sizes for health-related fitness outcomes (e.g., muscular strength, muscular endurance, cardiorespiratory endurance) were extracted from the same study. Traditional univariate meta-analytic approaches treat effect sizes within a study as independent, average them, and/or select only one per study (22). However, these approaches are problematic because they violate the assumption of independence among effect sizes, leading to inflated results, reduced statistical power, and/or limited research questions due to information loss. Thus, this study adopted a three-level meta-analytic model as an alternative because this model handles the dependency issue in effect sizes while preserving all information reported in a single study (22, 31).

By decomposing variance components across hierarchical levels, the three-level meta-analysis accounts for the sampling variance of effect sizes (Level 1), within-study heterogeneity among effect sizes extracted from the same study (Level 2), and between-study heterogeneity across different studies (Level 3) (22, 31). In the present study, Level 1 would represent the sampling variance of each effect size, determined by the participant sample size for fitness outcomes. Level 2 would capture the variance among multiple effect sizes, such as muscular strength, endurance, and BMI, extracted from the same study. Level 3 would indicate the variance across different independent studies, attributable to study-level features, such as school level, gender, and intervention characteristics.

Statistical analyses were conducted using the rma.mv function of the metafor package in R (v. 4.5.0). At least two studies measuring the same outcome were necessary to establish statistical reliability. A random-effects model was used to estimate the overall effect size, which allows for the variances at Level 2 and Level 3, thereby enabling unconditional inference in similar contexts (22). For the moderator analysis, however, a mixed-effects meta-regression model was employed because fixed moderators as identified in the data collection process were incorporated into the existing random-effects model, combining fixed effects with random variance components to ensure methodological rigor (30).

Effect sizes (Hedges' *g*) were interpreted using the benchmarks of Cohen (32) with interval boundaries: small (0.20 ≤ *g* ≤ 0.49), moderate (0.50 ≤ *g* ≤ 0.79), and large (*g* ≥ 0.80). As the thresholds are not absolute cut-offs but relative benchmarks (32), effect sizes were flexibly interpreted if they were in close proximity to a threshold. Heterogeneity across the effect sizes was determined by calculating Cochran's *Q* and $I^{2}$, while within- (Level 2) and between-study variance (Level 3) were determined by computing $I^{2}$, $\tau^{2}$, and the log-likelihood ratio test (LRT). The statistically significant *Q* statistics indicated potential heterogeneity. As the proportion of the total variation of the effect sizes, $I^{2}$ was interpreted as low (< 25%), substantial (25%–50%), and considerable (> 75%) heterogeneity. A significant LRT result (*χ^2^*, *p* < 0.05) indicates significant heterogeneity if $\tau^{2}$  > 0 as well as a rationale for using the three-level meta-analysis (22, 33). To determine whether study characteristics predicted the intervention effect, moderator analyses were conducted using a multivariate meta-regression method. Two sensitivity analyses were conducted: the first assessed the impact of the assumed pre-post correlations (i.e., *r* = 0.3, 0.5, 0.7) on selecting a correlation to calculate variance; and the second examined the impact of a single effect size on the overall effect size using a leave-one-out method to identify significant outliers. Publication bias was tested using a funnel plot and Egger's regression (30).

## 3 Results

### 3.1 Study selection process

**Figure 1:**
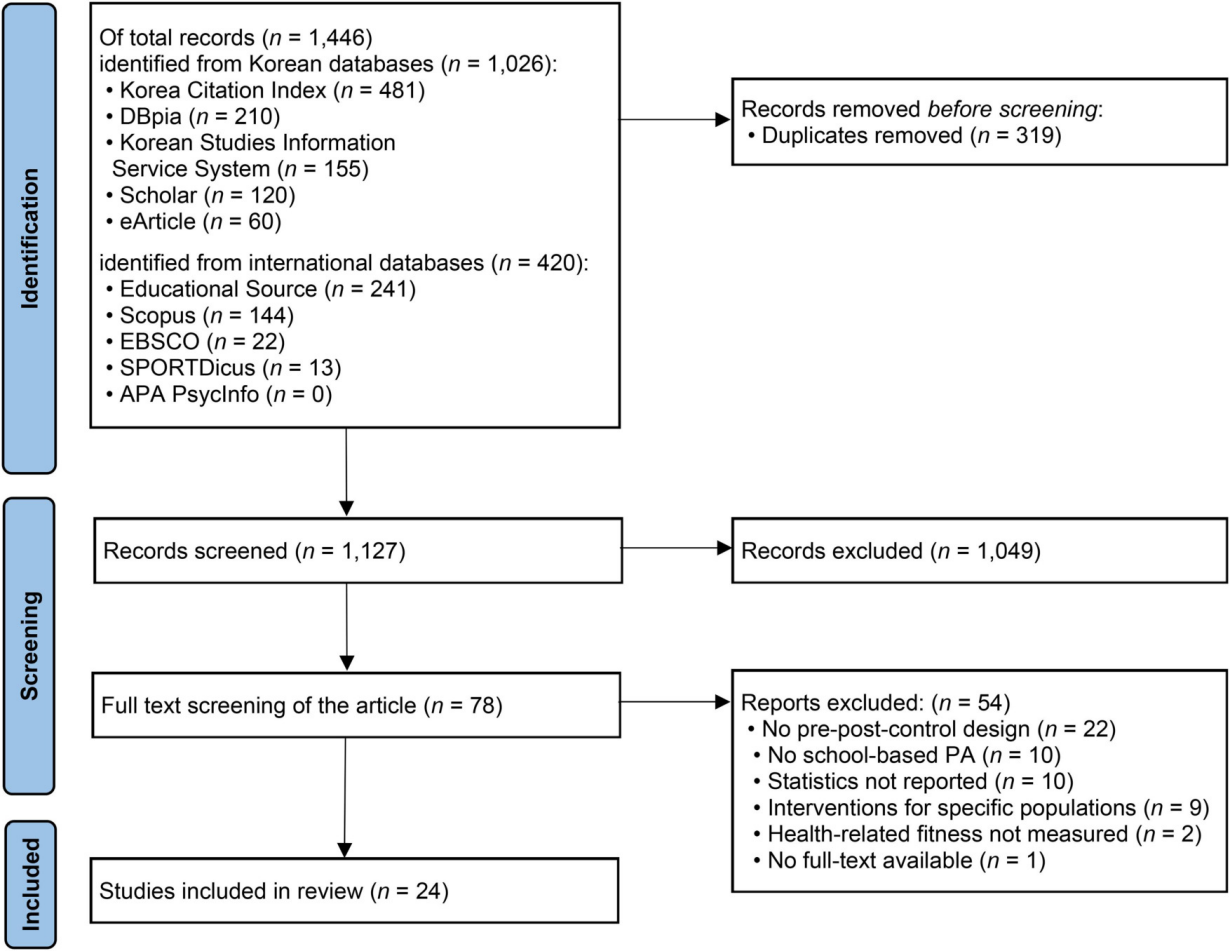
Flow diagram of literature search process. Reprinted with permission from The PRISMA 2020 statement: an updated guideline for reporting systematic reviews by Page, M. J., McKenzie, J. E., Bossuyt, P. M., Boutron, I., Hoffmann, T. C., Mulrow, C. D., & Whiting, P. (2021), Licensed under Creative Commons Attribution (CC BY 4.0), http://dx.doi.org/10.1136/bmj.n71

The result of the literature screening process is presented in Figure 1. The initial database search retrieved 1,446 from the Korean databases and 420 from the international databases. After removing duplicates (*n* = 319), 1,127 remained, of which 1,049 were excluded through screening of the title and abstract. The remaining 78 articles were full-text reviewed, of which 54 were excluded because of ineligibility as follows: no pre-post-control design (*n* = 22), no school-based PA interventions (*n* = 10), statistics not reported (*n* = 10), interventions for specific populations, such as those with disability or physical illness (*n* = 9), health-related fitness not measured (*n* = 2), and no full-text available (*n* = 1). This process, in turn, yielded 24 articles.

### 3.2 Characteristics of the intervention studies

Supplementary Table presents the characteristics of the included studies. They were implemented in elementary school (*n* = 14), middle school (*n* = 6), high school (*n* = 3), and a combination of elementary and middle school (*n* = 1). The total number of participants was 1,373 (*n* = 688, *M* = 28.67, *SD* = 22.29 in the experimental group, *n* = 685, *M* = 28.54, *SD* = 21.90 in the control group). The participants' gender was reported as all male and female in 13 studies, male-only in 10 studies, and female-only in one; among these, a study (34) provided outcomes separately by gender. Most interventions (*n* = 17) employed a non-randomized design, while seven adopted a randomized controlled design. The interventions were structured as warm-up stretching, main activity, and cool-down stretching, adopting a recreational approach (*n* = 14), a public health approach (*n* = 8), or an educational approach (*n* = 2). Half of the studies (*n* = 12) designed interventions based on rigorous physiological prescriptions, such as those based on % HRR. In contrast, the remaining studies (*n* = 12) did not establish specific intensity thresholds; instead, they nominally described the intensity or relied on the inherent structural characteristics of the activities. Interventions lasted an average of 15.25 weeks (*SD* = 10.35), ranging from 8 weeks to a year (two school semesters); included an average of 2.80 sessions per week (*SD* = 1.22), ranging from one per week to daily (five days per week); and averaged 48.43 min per session (*SD* = 16.63), ranging from 9 to 100 min.

### 3.3 Risk of bias assessment results

**Figure 2:**
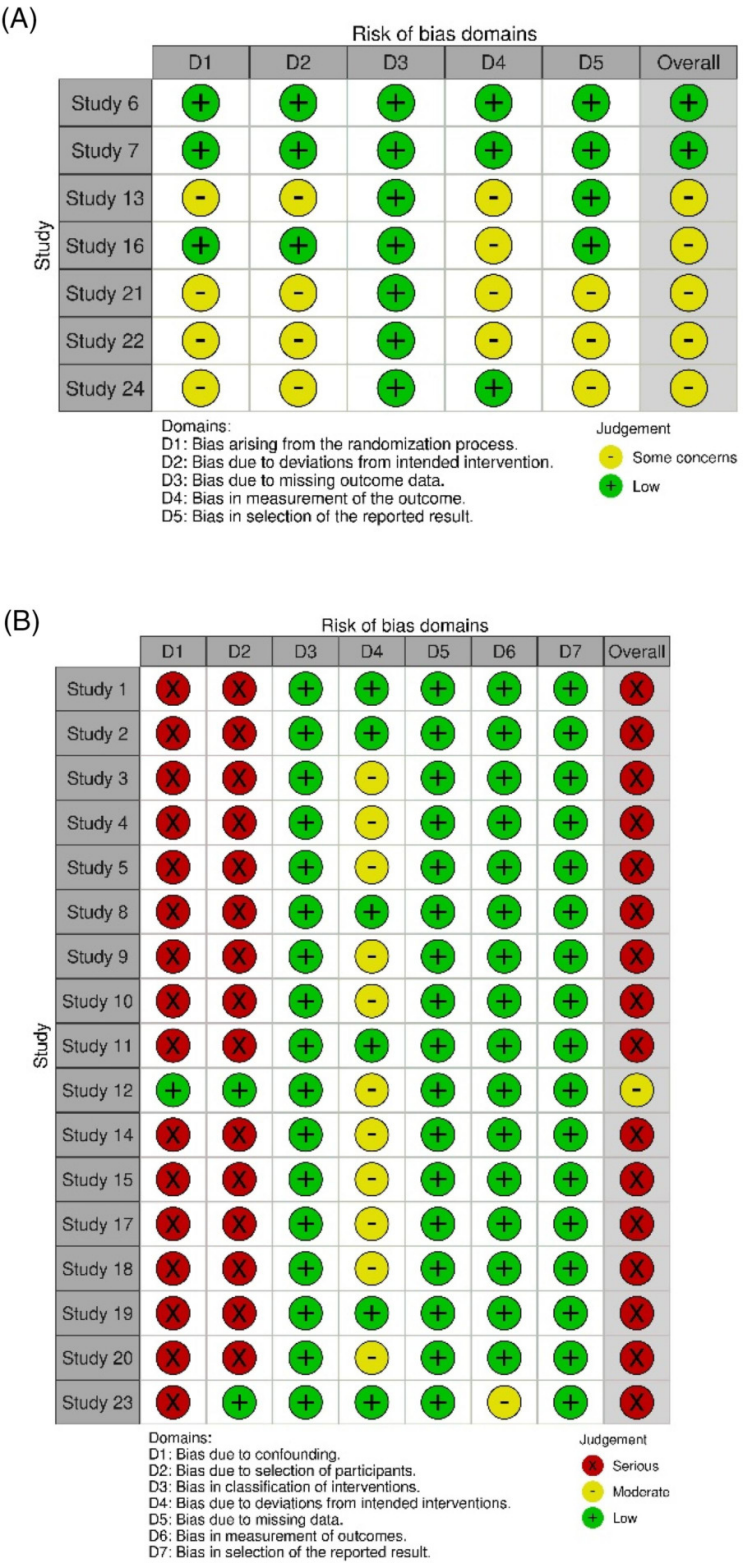
**(A)** Assessment result of risk of bias in randomized interventions. **(B)** Assessment result of risk of bias in non-randomized interventions.

The results of the risk of bias assessment were illustrated using RobVis (35). Figure 2 (A) displays the overall assessment results of RoB 2 as 'some concerns' in five studies, while ‘low risk' in two. Figure 2 (B) exhibits for ROBINS-I that most of the studies were assessed as 'serious' due to unmeasured, unknown confounders in Domain 1, as well as participant selection in Domain 2. These serious risks are often acknowledged in non-randomized intervention studies and school contexts; excluding these studies would result in a significant loss of real-world evidence (external validity). Therefore, this study decided to include the studies to provide the best available evidence in the meta-analysis.

### 3.4 Overall effect

The overall effect of the school-based PA interventions on health-related fitness and BMI was estimated using a three-level meta-analysis to account for the dependency of multiple effect sizes. A total of 114 effect sizes (Level 2) were nested within studies (Level 3) by clustering all effect sizes extracted from each study into shared variance components. To ensure transparency, Supplementary Table displays the number of effect sizes per study, ranging from 1 to 10. The three-level meta-analysis estimated a small-to-moderate effect size (Hedges' *g* = 0.33, *SE* = 0.05, *p* < 0.001).

Substantial heterogeneity was found at Level 2, with an estimated $\tau^{2}$ of 0.08 ($I^{2}$ = 46.28%), which was statistically significant (LRT=25.52, *p* < 0.001). No significant heterogeneity was observed in Level 3 ($\tau^{2}$ = 0.02, $I^{2}$  = 8.47%) with an LRT of 1.18 (*p* = 0.28). These specific variance components demonstrated that this three-level meta-analysis captured and partitioned the dependency among effect sizes. This approach provided a direct statistical test (e.g., LRT) to confirm that the within-study variability was the primary source of heterogeneity, which differs from robust variance estimation (RVE) and multivariate meta-analysis. While those methods account for dependency among effect sizes, they are limited in identifying the specific source of variance. Thus, the use of the three-level meta-analysis was justified as appropriate to fulfill the purpose of this study with methodological rigor.

### 3.5 Outcome-specific effect sizes

**Figure 3:**
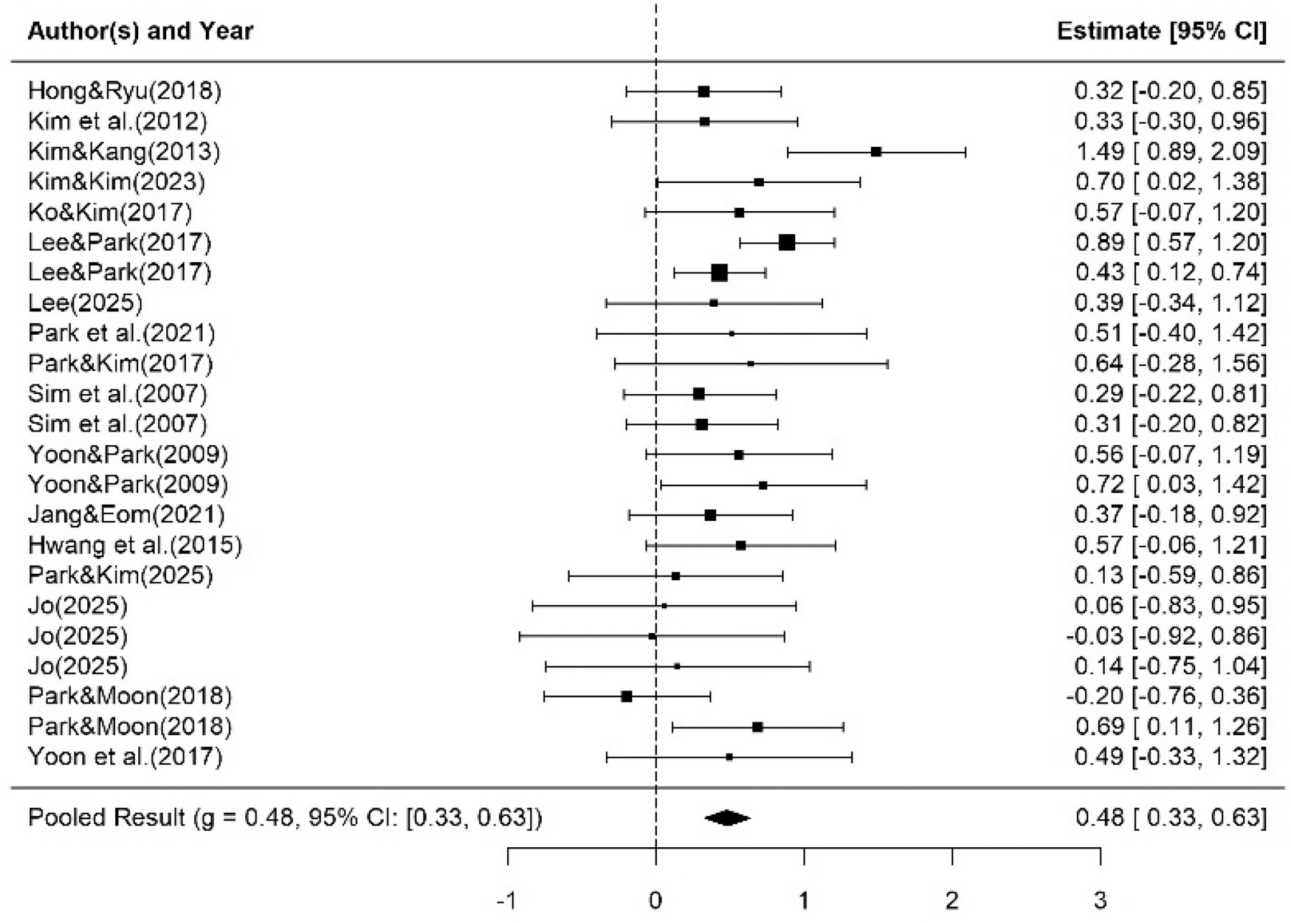
Forest plot for muscular endurance.

**Figure 4:**
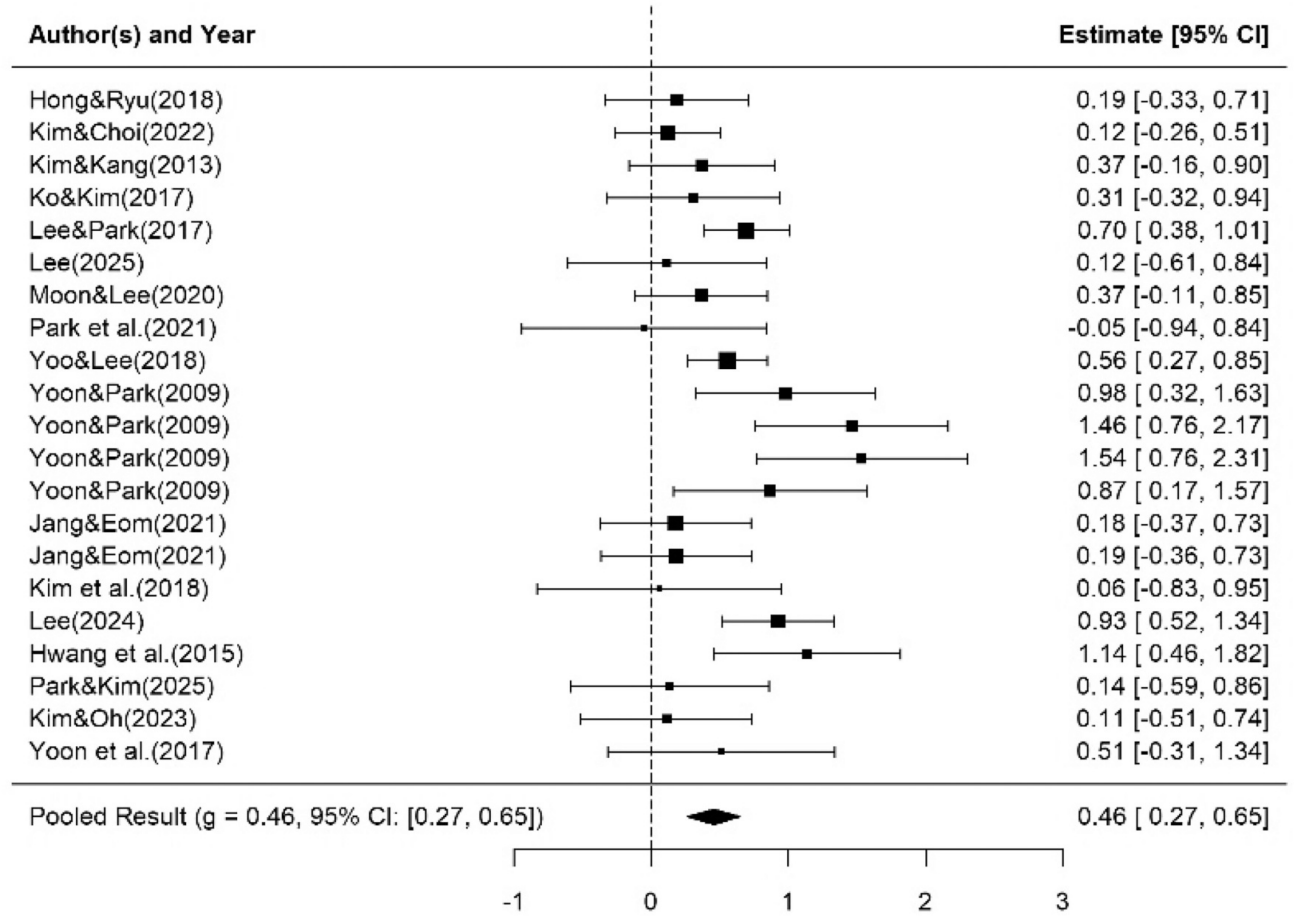
Forest plot for muscular strength.

**Figure 5:**
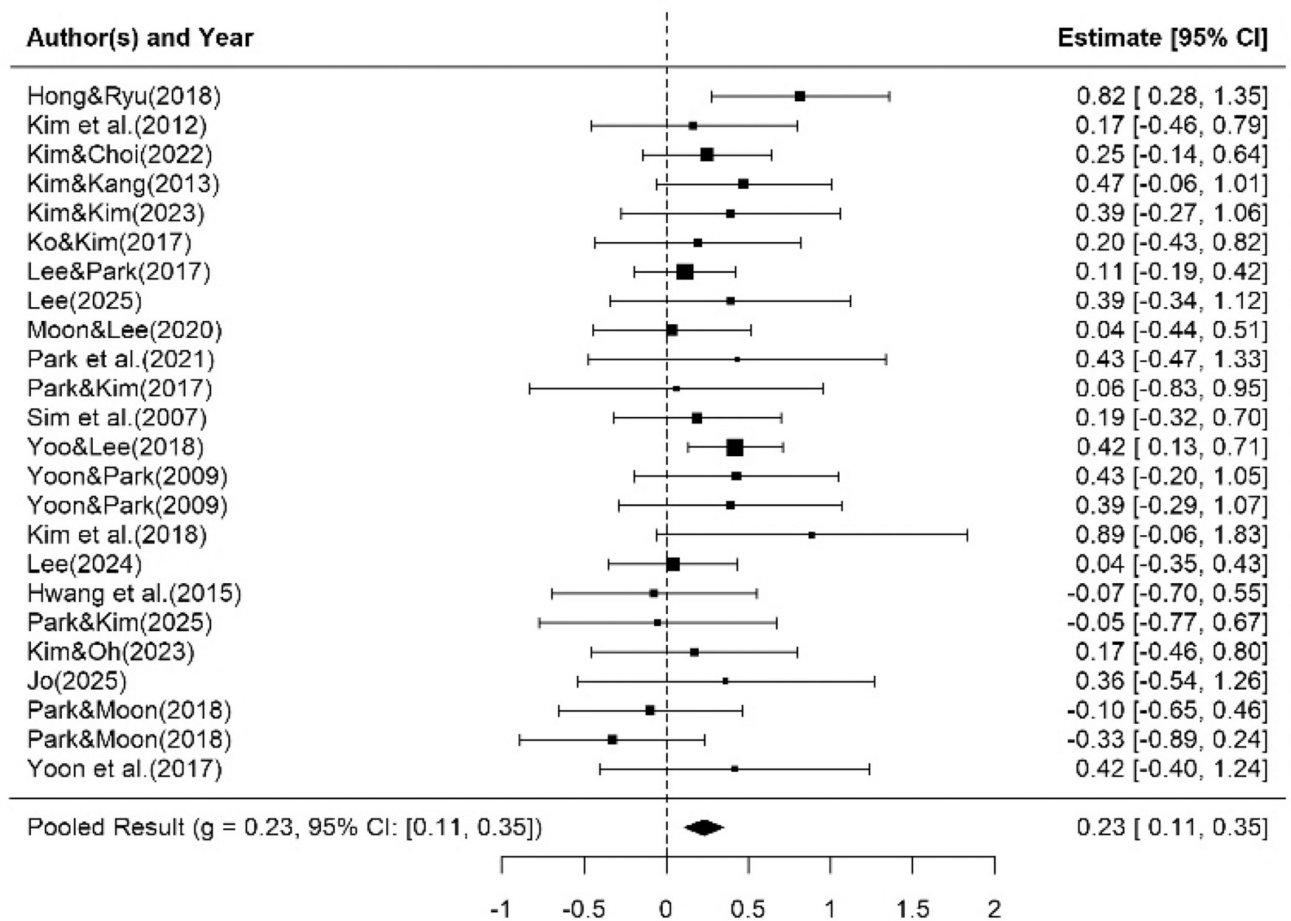
Forest plot for flexibility.

**Figure 6:**
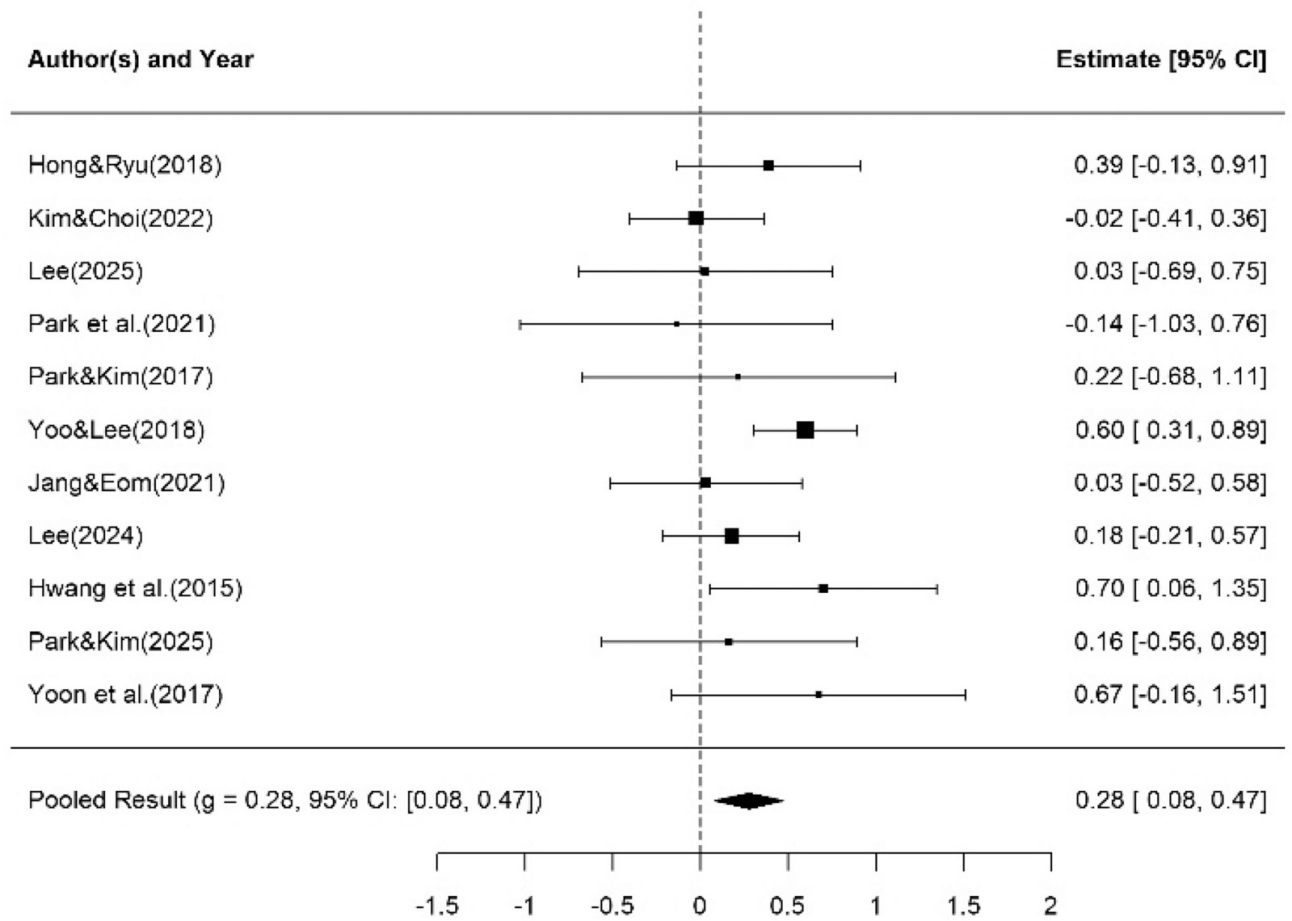
Forest plot BMI.

**Figure 7:**
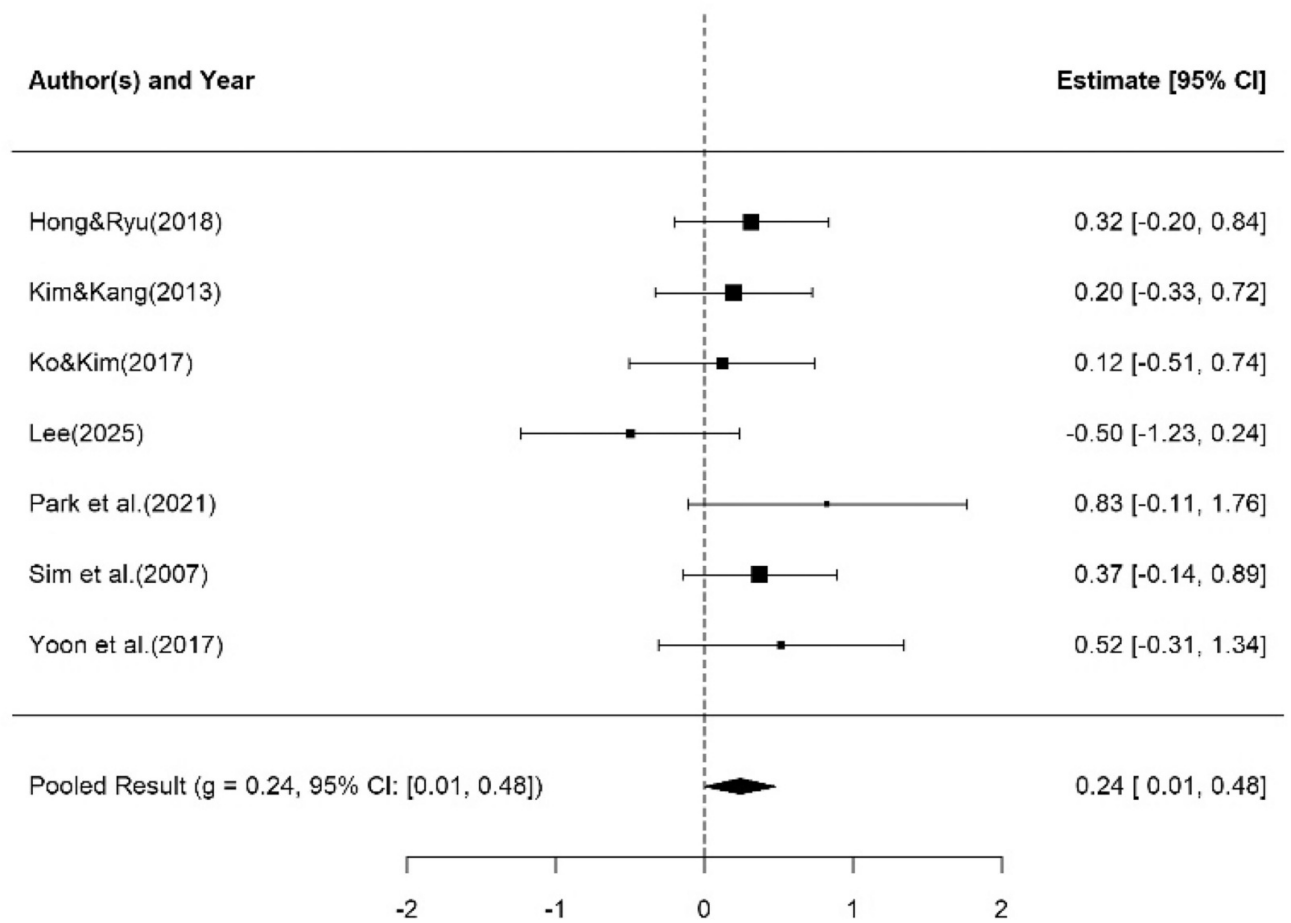
Forest plot for body fat %.

**Figure 8:**
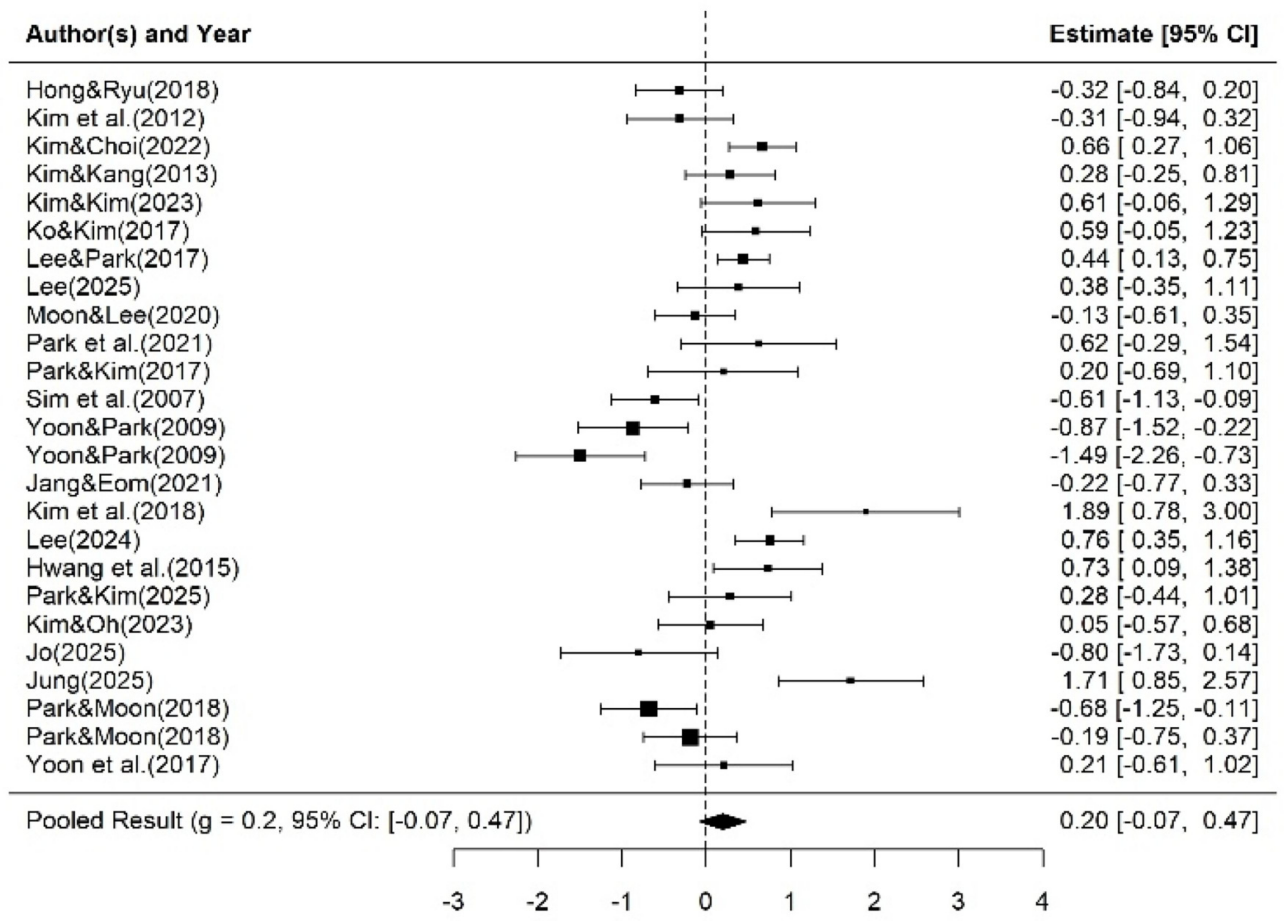
Forest plot for cardiorespiratory endurance.

Table 2 presents the number of the outcome-specific effect sizes nested in their respective intervention studies. Three effect sizes (i.e., muscle mass, fat mass, and lean mass) were excluded because each was reported in only one study. The remaining 111 effect sizes were used in the same three-level meta-analysis to maintain consistency in accounting for potential within-study dependency (Level 2). Figures 3–8 indicate forest plots for each outcome-specific effect size across the intervention studies. As seen in Table 2, significant outcomes were observed in muscular endurance (Hedges' *g* = 0.48, *p* < 0.001), muscular strength (Hedges' *g* = 0.46, *p* < 0.001), flexibility (Hedges' *g* = 0.23, *p* < 0.001), BMI (Hedges' *g* = 0.28, *p* = 0.01), and body fat % (Hedges' *g* = 0.24, *p* = 0.04). Cardiorespiratory endurance did not show a statistically significant change (*p* > 0.05). Among these outcomes, only muscular strength demonstrated significant heterogeneity (*Q*_(*df*_ *_=_* _20)_ = 40.8, *p* < 0.001), which could be attributed to between-study variance (Level 3), with estimated variances of $\tau^{2}$  = 0.08, $I^{2}$  = 53.09%, and a significant LRT of 5.79 (*p* = 0.02). Further analysis of the variance was not conducted to minimize the risk of inflating Type 1 error.

**Table 2:** Outcome-specific effect sizes.

Variable	*k_ES_*	*k_studies_*	*ES*	*SE*	±95% CI	*P*
Muscular endurance	23	17	0.48	0.08	[0.33, 0.63]	< 0.001
Muscular strength	21	17	0.46	0.10	[0.27, 0.65]	< 0.001
Flexibility	24	22	0.23	0.06	[0.11, 0.35]	< 0.001
BMI	11	11	0.28	0.10	[0.08, 0.47]	.01
Body fat %	7	7	0.24	0.12	[0.01, 0.48]	0.04
Cardiorespiratory endurance	25	23	0.20	0.14	[−0.07, 0.15]	0.15

### 3.6 Moderator analysis

In this study, six moderators were identified: school level, gender, intervention type, approach, intensity, and weekly duration (min/week). A multivariate meta-regression moderator analysis indicated that most moderators were not significant (*p* > 0.05). Only weekly duration (min/week) emerged as a significant moderator (*QM*_(*df*_ *_=_* _2)_ = 5.90, *p* = 0.04). Specifically, the 100–150 min/week dose was the most beneficial for improving students' health-related fitness compared with the <100 min/week and the >150 min/week groups (*β* = 0.28, *SE* = 0.12, *p* = 0.02). The >150 min/week group showed no significant difference from either the moderate group (*p* = 0.50) or the low group (*p* = 0.07).

### 3.7 Sensitivity analysis and publication bias

**Figure 9:**
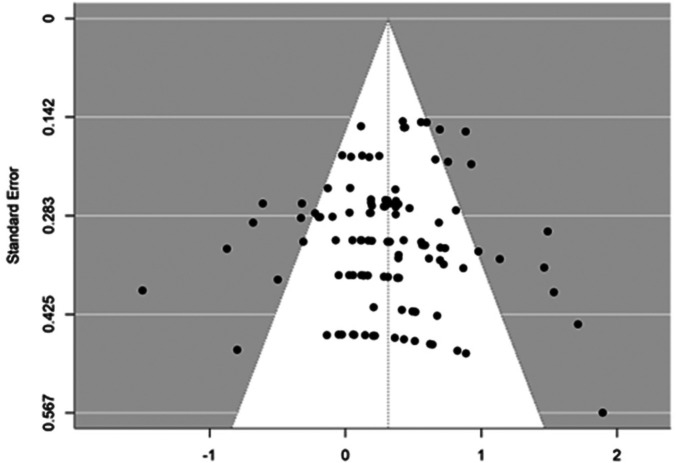
Funnel plot.

Sensitivity analysis of the assumed pre-post correlations (i.e., *r* = 0.3, 0.5, 0.7) exhibited consistency in effect sizes across *rs* (Hedges' *g* = 0.32, 0.31, 0.31, respectively), establishing a rationale for using *r* = 0.5 (27). The leave-one-out method for sensitivity analysis indicated the overall effect size ranged from 0.30 to 0.34 when any single study was removed. This result supports the robustness of the estimated overall effect size, with no evident outliers. For publication bias, Figure 9 displays a symmetric distribution of the effect sizes. The Egger's regression tests resulted in (*z* = 0.42, *p* = 0.68), suggesting that there was no evidence of substantial publication bias.

## 4 Discussion

The purpose of this study was to determine the effectiveness of school-based PA interventions on health-related fitness and BMI in South Korea. A three-level meta-analysis demonstrated a small-to-moderate effect. The observed difference in the effect size was evident within studies (Level 2) rather than between studies (Level 3). This difference suggests that the effectiveness of the interventions was relatively consistent across studies, whereas it varied in specific outcomes. In particular, muscular endurance and strength exhibited moderate effects; flexibility, BMI, and body fat % indicated relatively small effects; but cardiorespiratory endurance did not show statistically significant improvements. As expected in the negligible heterogeneity at Level 3, most moderators, including school level, gender, intervention type, intervention approach, and PA intensity, did not significantly explain differences in the effect size, while weekly duration emerged as a statistically significant moderator. These findings provide a potential benchmark for future intervention studies. However, caution should be taken in interpreting the findings due to methodological concerns.

Muscular endurance and strength exhibited moderate effects. These findings suggest that different PA programs in Korean schools are likely to be beneficial for improving muscular endurance and strength in children and adolescents, consistent with international systematic reviews and meta-analyses (18, 19). The school PA programs lasting an average of 15.85 weeks might lead to refinements in muscle architecture, such as enhanced musculotendinous stiffness, thereby facilitating muscular endurance and strength (36). Although the literature has recommended that PA interventions should be specifically designed to foster muscular endurance and strength, such as plyometric resistance training (37) and HIIT (19, 20), the findings of this study imply that simply engaging in school-based PA interventions may be sufficient for Korean children and adolescents to enhance these outcomes, even without such an explicit focus. This interpretation is strongly supported by the results of moderator analysis, which showed insignificant moderators, such as intervention types and approaches. This lack of moderating effects seem to reinforce the idea that, in contexts where children and adolescents are highly sedentary and physically inactive, such as South Korea (9), providing opportunities for PA participation is a paramount stimulus for muscular adaptation.

The effect of school-based interventions on flexibility was small but significant. This finding provides empirical evidence in the literature, considering that flexibility has rarely been measured in international school-based PA intervention research (19). The observed improvement in flexibility may be explained by physiological mechanisms that reduce musculotendinous stiffness and increase pliability. Childhood and adolescence are developmental periods where tendon structures are relatively compliant, making them highly responsive to even general physical stimuli (38). The PA interventions might enable the participants to involve a consistent stimulus and a greater range of motion during warm-ups, main activities, and cool-downs, thereby improving the viscoelastic properties of connective tissues and increasing stretch tolerance.

BMI and body fat % exhibited small but significant decreases. Consistent with these findings, previous studies have demonstrated that different PA interventions for children and adolescents, such as HIIT and aerobic activities, contributed to decreases in BMI and body composition, such as body fat % (17, 18). In this study, by engaging in school-based PA interventions, the students might facilitate caloric expenditure over caloric consumption, contributing to the reduction in adiposity (7). School-based PA interventions, thus, may be beneficial for preventing pediatric obesity among Korean children and adolescents. However, the specific effects on other body composition indicators, such as muscle mass and lean mass, were not identified because they were excluded from the current meta-analysis due to the lack of information reported, which limits the depth of our interpretation regarding the qualitative changes in body tissues. Despite this lack of the mass-based data, the significant improvements in muscular endurance and strength observed in this study imply that the interventions may induce positive functional adaptations. These findings suggest that decreases in BMI and body fat % likely reflects a healthy shift in body composition.

An unexpected finding was the insignificant improvement in cardiorespiratory endurance. Previous studies have confirmed that school-based PA interventions across countries help children and adolescents foster cardiorespiratory endurance (16–18). In addition, cardiorespiratory endurance, muscular endurance, and muscular strength are often positively correlated (39), suggesting that improvements in one often accompany the other. One potential explanation for this inconsistency is that the included interventions might be sufficient to stimulate neuromuscular adaptations in muscular fitness, which are highly responsive to diverse stimuli (37), but were unlikely to reach the specific metabolic threshold for cardiorespiratory endurance adaptation in the Korean school context. This failure to reach the metabolic threshold may be attributed to the lack of intervention design in detail or description in the included studies. Empirically, a shift in cardiorespiratory endurance is influenced by overload in intensity, stimulation, duration, and progression (16, 18, 19). However, in the present study, half of the studies did not develop the programs based on rigorous physiological prescriptions such as % HRR. In addition, three studies (24%) did not clearly describe the weekly duration. Consequently, the null finding for cardiorespiratory endurance may reflect inconsistencies in exercise prescription or a lack of description of interventions, which should be addressed in future research.

Moderator analysis exhibited that most of the moderators (i.e., school level, gender, intervention type, intervention approach, and PA intensity) were not statistically significant. These findings appear to reflect the unique socio-cultural context of South Korea, characterized by highly standardized school curricula and school operations at a national level, and extreme academic competition and the college entrance exam, marginalizing school PA programs, such as physical education, with limited facilities and administrative hurdles (40–44). In this context, PA programs in schools seem to be mostly designed based on recreational or public health approaches, implying that Korean school-based PA interventions generally prioritize enjoyable experiences or MVPA. This standardized environment appears to have constrained variability across the intervention characteristics, which may explain the non-significant moderating effects. According to Ennis (45), there are three approaches to PA programs: a recreational approach aimed at providing enjoyable activities; a public health approach designed to promote PA participation and meet PA guidelines; and an educational approach. In particular, educational approach-based PA programs enhance participants' knowledge of health-related fitness and promote PA participation. The first two approaches can contribute to positive changes in fitness outcomes. Still, the absence of the educational approach may limit long-term effectiveness due to a lack of knowledge and understanding of health-related fitness and PA (45). Incorporating an educational approach into these PA programs is likely to enhance not only immediate health-related fitness outcomes but also the knowledge and skills necessary for children and adolescents to engage in lifelong PA (46). Further intervention studies should be designed and tested based on the educational approach.

Weekly duration was a sole significant moderator. In particular, the 100–150 min/week dose is likely to have the most favorable effects on health-related fitness and BMI. While empirical evidence has supported a proportional dose–response relationship, recommending at least 3 sessions per week (each lasting ≥ 30 min) (36), the findings of this study seem to suggest a potential context-specific threshold. Specifically, for Korean children and adolescents, whose PA levels are extremely low (9), the 100–150 min/week dose may be a practical range to trigger measurable improvements in health-related fitness and BMI within their intensive academic schedules (25). However, caution is required when interpreting the findings. To determine the optimal duration of interventions for Korean children and adolescents, interactions among other factors, such as program design (e.g., intensity control, session structure, exercise modality, progression) and participant characteristics (e.g., baseline fitness, adherence, motivation), should be considered together. In addition, as described above, the exclusion of three studies due to missing duration data limits the generalizability of this finding. Given that meeting the WHO PA guidelines recommending at least 60 min of daily MVPA (8) is often unfeasible in the Korean school context, this 100–150 min/week range may serve as a content-specific, preliminary benchmark for realistic intervention planning.

However, several methodological concerns about the included studies should be cautiously addressed when interpreting the results. First, the sample sizes in each study were small, which limits statistical power and hinder the identification of true effects. Second, the moderating effect of gender was not statistically significant primarily due to the unbalanced sample size, which results in a lack of evidence to conclude that the interventions were effective for all females and males. According to the Korea Disease Control and Prevention Agency (47), PA participation rates among children and adolescents differ substantially between females and males. The rates for PA as well as muscle-strengthening activity have been consistently lower in females than males over the last 10 years. These differences in participation rates by gender may offset the effectiveness of the interventions. Thus, the insignificant moderating effect of gender should not be concluded until the intervention effectiveness on females' health-related fitness and BMI is thoroughly examined. Third, the overall risk of bias in the non-randomized studies was assessed as 'serious' primarily due to judgments in Domains 1 and 2. Most sources of the judgments were a lack of controlling potential confounders and the pre-selection of the participants. Although these judgments reflect the common nature of non-randomized studies and school contexts, they might lead to the insignificance of moderator analysis, limiting generalizability of the findings (28). Lastly, there might be potential factors that have not been explored due to the limited information available in this study. Fidelity of implementation, for example, can be a critical strategy for clarifying the effectiveness of the interventions. Loflin (48) found that the more closely physical education teachers adhere to the fidelity of implementation of provided school-based PA programs, the more likely children and adolescents are to participate in in-class PA and enhance learning outcomes, suggesting a potential influence of fidelity of implementation on health-related fitness and BMI.

## 5 Strengths and limitations

This three-level meta-analysis has several strengths. First, this is the first study to synthesize and meta-analyze the effectiveness of school-based PA interventions on health-related fitness and BMI in South Korea, providing insights into context specificity in the intervention effectiveness. Second, by adopting a three-level meta-analytic model, this study addressed the issues of dependency, inflation, and overconfidence in effect sizes while preserving all effect sizes reported in the included studies. Lastly, rigorous adherence to the risk of bias assessment criteria ensured methodological transparency and the credibility of the findings. However, there were also several limitations. As addressed above, the small sample size, unbalanced sample size by gender, the absence of the rigorous physiological prescriptions, missing information of weekly duration, and the serious risk of bias constrain the validity of the findings. In addition, generalizability and applicability is limited across countries, as this study included only interventions conducted in the Korean context, reflecting a context-specific nature of PA interventions. It is recommended that future research examine how cultural differences lead to variations in the effects of school-based PA interventions.

## 6 Conclusions and implications

The purpose of this study was to determine the effectiveness of school-based PA interventions on health-related fitness and BMI in South Korea. Adopting a three-level meta-analytic model, this study found a small-to-moderate intervention effect. Specifically, muscular endurance and strength appeared to exhibit moderate effects, and flexibility, BMI, and body fat % indicated small effects, but cardiorespiratory endurance did not have a significant effect. Moderator analysis demonstrated that only weekly duration (100–150 min/week) were statistically significant, whereas the others, including school level, gender, intervention type, intervention approach, and PA intensity, were not. Although these findings provide insight into school-based PA program design, they should be cautiously interpreted due to the small sample size and quality of the interventions, limiting generalizability of the findings.

The findings of this study offer at least four critical implications for practice and future research. First, the design-to-practice gap can be minimized by providing objectively prescribed PA intensity. Future studies should empirically verify precise intensity criteria structurally embedded and operationalized within the intervention design. Second, complex interactions among program design (e.g., intensity control, session structure, exercise modality, progression) and participant characteristics (e.g., baseline fitness, adherence, motivation), should be addressed together in developing a school-based PA intervention to confirm the optimal weekly dose. Third, more randomized controlled trials with low risk of bias should be conducted to establish validity of the finding. Last, future school-based PA programs can be designed based on an educational approach to help Korean children and adolescents acquire the knowledge needed for lifelong PA and health-related fitness.

## Data Availability

The raw data supporting the conclusions of this article will be made available by the authors, without undue reservation.

## References

[1] Muyulema SL Carpio-Arias TV Verdezoto N Lara VE Manzano AS Pulgar H. Worldwide trends in childhood overweight and obesity over the last 20 years. Clin Nutr ESPEN. (2025) 1(65):453–60. 10.1016/j.clnesp.2024.12.013 39709095

[2] World Health Organization. Obesity and overweight*.* (2025). Available online at: https://www.who.int/news-room/fact-sheets/detail/obesity-and-overweight#:∼:text=lived%20in%20Asia.-,Over%20390%20million%20children%20and%20adolescents%20aged%205%E2%80%9319%20years,1990%20to%2020%25%20in%2020 (Assessed October 15, 2025).

[3] Hagman E Danielsson P Elimam A Marcus C. The effect of weight loss and weight gain on blood pressure in children and adolescents with obesity. Int J Obes. (2019) 43(10):1988–94. 10.1038/s41366-019-0384-2 31152153

[4] Puhl RM Lessard LM. Weight stigma in youth: prevalence, consequences, and considerations for clinical practice. Curr Obes Rep. (2020) 9(4):402–11. 10.1007/s13679-020-00408-8 33079337

[5] Schwartz MW Seeley RJ Zeltser LM Drewnowski A Ravussin E Redman LM. Obesity pathogenesis: an endocrine society scientific statement. Endocr Rev. (2017) 38:267–96. 10.1210/er.2017-00111 28898979 PMC5546881

[6] Salam RA Padhani ZA Das JK Shaikh AY Hoodbhoy Z Jeelani SM. Effects of lifestyle modification interventions to prevent and manage child and adolescent obesity: a systematic review and meta-analysis. Nutr. (2020) 12(8):2208. 10.3390/nu12082208 PMC7468898 32722112

[7] Stoner L Rowlands D Morrison A Credeur D Hamlin M Gaffney K. Efficacy of exercise intervention for weight loss in overweight and obese adolescents: meta-analysis and implications. Sports Med. (2016) 46(11):1737–51. 10.1038/sj.ijo.0803286 27139723

[8] World Health Organization. *WHO Guidelines on PA and sedentary behaviour**.* (2020). Available online at: https://apps.who.int/iris/handle/10665/336656 (Assessed October 17, 2025).

[9] Guthold R Stevens GA Riley LM Bull FC. Global trends in insufficient PA among adolescents: a pooled analysis of 298 population-based surveys with 1·6 million participants. Lancet Child Adolesc Health. (2020) 4:23–35. 10.1016/S2352-4642(19)30323-2 31761562 PMC6919336

[10] Tomkinson GR Lang JJ Tremblay MS. Temporal trends in the cardiorespiratory fitness of children and adolescents representing 19 high-income and upper middle-income countries between 1981 and 2014. Br J Sports Med. (2019) 53(8):478–86. 10.1136/bjsports-2017-097982 29084727

[11] Kasović M Oreški A Vespalec T Jenčíková K Štefan L. Tracking of health-related physical fitness in adolescent girls: a 3-year follow-up study. BMC pediatr. (2022) 22(1):236. 10.1186/s12887-022-03305-2 35488259 PMC9052589

[12] Weedon BD Liu F Mahmoud W Burden SJ Whaymand L Esser P. Declining fitness and physical education lessons in UK adolescents. BMJ Open Sport Exerc Med. (2022) 18(8):e001165. 10.1136/bmjsem-2021-001165 PMC8768926 35127132

[13] Society of Health and Physical Educators America. CSPAP comprehensive school PA programs: what *is CASPAP?* Available online at: https://www.shapeamerica.org/cspap/what.aspx (Assessed October 17, 2025).

[14] Eather N Morgan PJ Lubans DR. Improving the fitness and PA levels of primary school children: results of the fit-4-fun group randomized controlled trial. Prev Med. (2013) 56:12–9. 10.1016/j.ypmed.2012.10.019 23107669

[15] Lee S. Changes in PAPS and body composition of elementary school student after participating in school kinball sports club. J Creat Info Cult. (2025) 11(1):135–46. 10.32823/jcic.11.1.202502.135

[16] Liu J Zhu L Su Y. Comparative effectiveness of high-intensity interval training and moderate-intensity continuous training for cardiometabolic risk factors and cardiorespiratory fitness in childhood obesity: a meta-analysis of randomized controlled trials. Front Pysiol. (2020) 11:214. 10.3389/fphys.2020.00214 PMC7145974 32308627

[17] Neil-Sztramko SE Caldwell H Dobbins M. School-based PA programs for promoting PA and fitness in children and adolescents aged 6 to 18. Cochrane Database Syst Rev. (2021) 9:CD007651. 10.1002/14651858.CD007651.pub3 34555181 PMC8459921

[18] Wu J Yang Y Yu H Li L Chen Y Sun Y. Comparative effectiveness of school-based exercise interventions on physical fitness in children and adolescents: a systematic review and network meta-analysis. Front Public Health. (2023) 11:1194779. 10.3389/fpubh.2023.1194779 37342273 PMC10278967

[19] Zhou X Li J Jiang X. Effects of different types of exercise intensity on improving health-related physical fitness in children and adolescents: a systematic review. Sci Rep. (2024) 14:14301. 10.1038/s41598-024-64830-x 38906965 PMC11192957

[20] Eddolls WT McNarry MA Stratton G Winn CO Mackintosh KA. High-intensity interval training interventions in children and adolescents: a systematic review. Sports Med. (2017) 47(11):2363–74. 10.1007/s40279-017-0753-8 28643209 PMC5633633

[21] Delgado-Floody P Latorre-Román P Jerez-Mayorga D Caamano-Navarrete F García-Pinillos F. Feasibility of incorporating high-intensity interval training into physical education programs to improve body composition and cardiorespiratory capacity of overweight and obese children: a systematic review. J Exerc Sci Fit. (2019) 17(2):35–40. 10.1016/j.jesf.2018.11.003 30740131 PMC6353718

[22] Cheung MWL. Modeling dependent effect sizes with three-level meta-analyses: a structural equation modeling approach. Psychol Methods. (2014) 19:211–29. 10.3389/fpsyg.2014.01521 23834422

[23] Bauman AE Reis RS Sallis JF Wells JC Loos RJ Martin BW. Correlates of physical activity: why are some people physically active and others not? Lancet. (2012) 380(9838):258–71. 10.1016/S0140-6736(12)60735-1 22818938

[24] Heath GW Parra DC Sarmiento OL Andersen LB Owen N Goenka S. Evidence-based intervention in physical activity: lessons from around the world. Lancet. (2012) 380(9838):272–81. 10.1016/S0140-6736(12)60816-2 22818939 PMC4978123

[25] Korean Statistical Information Service. *Private education participation rate by school level*. (2024). Available online at: https://kosis.kr/statHtml/statHtml.do?orgId=101&tblId=DT_1PE301&conn_path=I2&language=en (Assessed November 1, 2025).

[26] Page MJ McKenzie JE Bossuyt PM Boutron I Hoffmann TC Mulrow CD. The PRISMA 2020 statement: an updated guideline for reporting systematic reviews. Br Med J. (2021) 372:n71. 10.1136/bmj.n71 33782057 PMC8005924

[27] Higgins JPT Savović J Page MJ Elbers RG Sterne JAC. Chapter 8: Assessing risk of bias in a randomized trial. In: Higgins JPT Thomas J Chandler J Cumpston M Li T Page MJ, editors. Cochrane Handbook for Systematic Reviews of Interventions version 6.5. Cochrane (2024). https://www.cochrane.org/handbook

[28] Sterne JAC Hernán MA McAleenan A Reeves BC Higgins JPT. Chapter 25: assessing risk of bias in a non-randomized study. In: Higgins JPT Thomas J Chandler J Cumpston M Li T Page MJ, editors. Cochrane Handbook for Systematic Reviews of Interventions version 6.5. Cochrane (2024). https://www.cochrane.org/handbook

[29] Morris SB. Estimating effect sizes from pretest-posttest-control group designs. Organ Res Methods. (2008) 11:364–86. 10.1177/1094428106291059

[30] Borenstein M Hedges LV Higgins JP Rothstein HR. Introduction to Meta-analysis. Hoboken, NJ: John Wiley & Sons (2021).

[31] Van Den Noortgate W Onghena P. Multilevel meta-analysis: a comparison with traditional meta-analytical procedures. Educ Psychol Meas. (2003) 63:765–90. 10.1177/0013164403251027

[32] Cohen J. Statistical Power Analysis for the Behavioral Sciences. Hillsdale, MI: Erlbaum (1998).

[33] Higgins JP Thompson SG Deeks JJ Alstman DG. Measuring inconsistency in meta-analyses. Br Med J. (2003) 327:557–60. 10.1136/bmj.327.7414.557 12958120 PMC192859

[34] Yoon YJ Park JY. The effects of school sports club activities in elementary school students' Physical self description and changing health related components of physical fitness using jump-roping approval program. J Res Curr Inst. (2009) 13:133–51. 10.24231/rici.2009.13.1.133

[35] McGuinness LA Higgins JPT. Risk-of-bias VISualization (robvis): an R package and shiny web app for visualizing risk-of-bias assessments. Res Synth Methods. (2020) 12:55–61. 10.1002/jrsm.1411 32336025

[36] Suchomel TJ Nimphius S Bellon CR Stone MH. The importance of muscular strength: training considerations. Sports Med. (2018) 48(4):765–85. 10.1007/s40279-018-0862-z 29372481

[37] Cox A Fairclough SJ Kosteli MC Noonan RJ. Efficacy of school-based interventions for improving muscular fitness outcomes in adolescent boys: a systematic review and meta-analysis. Sports Med. (2020) 50(3):543–60. 10.1007/s40279-019-01215-5 31729638 PMC7018678

[38] Donti O Konrad A Panidi I Dinas PC Bogdanis GC. Is there a “window of opportunity” for flexibility development in youth? A systematic review with meta-analysis. Eur J Sport Sci. (2022) 8:88. 10.1186/s40798-022-00476-1 PMC9259532 35792993

[39] Evaristo S Moreira C Lopes L Oliveira A Abreu S Agostinis-Sobrinho C. Muscular fitness and cardiorespiratory fitness are associated with health-related quality of life: results from labmed physical activity study. J Exerc Sci Fit. (2019) 17(2):55–61. 10.1016/j.jesf.2019.01.002 30740134 PMC6353732

[40] Hong SW Rye JS. Effects of niche PA on the body composition and basic physical fitness of elementary school students. Korean Assoc Learn Cent Curr Inst. (2018) 18:461–79. 10.22251/jlcci.2018.18.23.461

[41] Kim BS Lee CS Oh YS. Effects of after-school PA programs on improvement of physical fitness for middle school male students. Educ Res Inst. (2012) 55:271–97. 10.17253/swueri.2012.55..009

[42] Kim JG Oh JS. The effects of circuit training with outdoor exercise equipment on the health-related physical fitness and physical self-efficacy of students in a low fitness level. J Korean Leis Sci. (2023) 14:141–51. 10.37408/kjls.2023.14.2.141

[43] Park JW Koh SH Ha SM Kim DY. The effects of soccer club activities as part of school sports on the health-related fitness, dopamine, and cortisol of boys in middle school. J Korean Leis Sci. (2021) 12:21–8. 10.37408/kjls.2021.12.3.21

[44] Sim JS Shon JH Yoo HS. The effect of soccer league program after school on physical fitness, physical self-concept, and academic achievement in high school students. Korea Coach Dev Cent. (2007) 9:115–26.

[45] Ennis CD. Educating students for a lifetime of physical activity: enhancing mindfulness, motivation, and meaning. Res Q Exerc Sport. (2017) 88:241–50. 10.1080/02701367.2017.1342495 28742426

[46] Chen A. Reconceptualizing Physical Education: A Curriculum Framework for Physical Literacy*.* New York: Routledge (2023).

[47] Korea Disease Control and Prevention Agency. *Statistics of the 20 youth risk behavior survey* (2024). Available online at: https://www.kdca.go.kr/yhs/yhs/main.do (Assessed November 1, 2025).

[48] Loflin JW. Relationship between teacher fidelity and physical education student outcomes. Phys Educ. (2015) 72:358–83. 10.18666/TPE-2015-V72-I5-7001

