# Supplementary material for: The effectiveness of school-based physical activity interventions on health-related fitness and BMI in South Korea: a three-level meta-analysis
Source: Front Pediatr. 2026 Jun 3;14:1849629. doi: 10.3389/fped.2026.1849629 (PMC13272293; doi:10.3389/fped.2026.1849629)
Supplement: Supplementary file 1 [file Datasheet1.docx]

Supplementary Table Characteristics of the included studies

| ID | Author | School level | Gender | n_  Exp | n_  Con | n_ ES | Outcome | Design | Types | Approach | Description of  interventions | Intensity (Target & Threshold) | Period | W_duration (min/week) | Analysis | |  |  |
| --- | --- | --- | --- | --- | --- | --- | --- | --- | --- | --- | --- | --- | --- | --- | --- | --- | --- | --- |
| 1 | Hong & Ryu (40) | Ele | F&M | 29 | 29 | 6 | BMI, Cardio, Fat %, Flex, M_end, M_str | Non-Ran | PA break | PH | Exp: Health-related fitness PA break during before school, during recess, during lunch, and after school. Activities included speed stacks, stretching, squats, planks, individual and group jump ropes, and walking. A self-check board was used in the classroom to encourage continuous, self-monitored participation.  Con: No PA break | Low-Mod (<60%HRmax​) | 16 weeks; daily; 31–41.5 min | 181.3 | Ind *t*-test, RM ANOVA | |  |  |
| 2 | Hwang et al. (49) | Ele | F&M | 20 | 20 | 5 | BMI, Cardio, Flex, M_end, M_str | Non-Ran | After-school PA | Rec | Exp: After-school Tchoukball program covering fundamental skills (e.g., short/middle/long passes, throw-ins, various angle shooting, and defending the drop zone), team tactical planning, and formal game play  Con: No after-school PA program | Mod (Nominal) | 12 weeks; 3 sessions per week; two 40-min sessions & one 80-min session | 160 | Dep *t*-test, ind *t*-test | |  |  |
| 3 | Jang &  Eom (50) | Ele | F&M | 26 | 26 | 5 | BMI, Cardio, M_end, M_str | Non-Ran | In and out of school PA | PH | Exp: Daily use of a wearable device devices to track daily steps and weekly activity volume in and out of school, with education sessions on exercise methods and the functions of the device in PE and school PA classes, often involving running, squats, lunges, planks, and stretching, along with home training guidance where students uploaded weekly activity volumes to a Padlet board for rewards  Con: Regular PE without wearing device | MVPA (Nominal) | 12 weeks; daily | - | Dep *t*-test, ind *t*-test | |  |  |
| 4 | Jo (51) | Ele | F&M | 10 | 10 | 5 | Cardio, Flex, M_end | Ran | After_school_PA | Rec | Exp: Music jump rope intervention divided into adaptation (basic steps), improvement (advanced skills, direction changes, and choreographic routine memorization) and application stages (double-unders and complex routine completion) Con: General PA without involving in the intervention (e.g., basic gymnastics, free jump rope) | Mod (RPE 11–13) | 12 weeks; 3 sessions per week; 60 min each | 180 | RM ANOVA, ANCOVA | |  |  |
| 5 | Jung (52) | Ele | F&M | 15 | 15 | 1 | Cardio | Ran | PE | PH | Exp: Structured PE class following interval running, a 6-station circuit training utilizing step boxes and medicine balls, small-group game play  Con: Regular PE with no intervention | MVPA (PRE 11–15) | 12 weeks; 3 sessions per week; 40 min each | 120 | RM ANOVA | |  |  |
| 6 | Kim et al. (41) | Mid | M | 20 | 20 | 3 | Cardio, Flex, M_end | Non-Ran | After-school PA | Rec | Exp: After-school PA program consisting of modified PA games, including soccer, jogging, ball games, badminton, and basketball with ball-stealing mini-games and small-group league matches to facilitate interest  Con: No after-school PA program | Vig (Nominal) | 12 weeks; 4 sessions per week; 45 min each | 180 | Ind *t*-test, RM ANOVA | |  |  |
| 7 | Kim et al. (53) | Mid | M | 10 | 10 | 3 | Cardio, M_end, M_str | Ran | After-school PA | Rec | Exp: After-school basketball program that progressed from basic body control, ball handling, passing, dribbling, and shooting at 40-60% HRR, to rebound games at 50-70% HRR, and finally man-to-man/zone defense and 5-on-5 full-court games at 50-80% HRR  Con: No after-school PA program | MVPA (40−80%HRR) | 12 weeks; 3 sessions per week; 60 min each | 180 | Ind *t*-test, RM ANOVA | |  |  |
| 8 | Kim & Choi (54) | Ele | F&M | 51 | 53 | 4 | BMI, Cardio, Flex, M_end, M_str | Non-Ran | PE | Rec | Exp: Game-based track-and-field program for kids in PE, including hurdle relays, speed ladder drills, cross hops, squat jumps, youth javelin throwing, accuracy throwing, and a complex relay called ‘Formula One’ (combining forward rolls, hurdles, and S-shaped runs).  Con: Regular PE | MVPA (RPE 13−15) | 12 weeks; 1 session per week; 40 min each | 40 | Ind *t*-test, RM ANOVA | |  |  |
| 9 | Kim & Kang (55) | Mid | F&M | 28 | 28 | 6 | Cardio, Fat%. Flex, M_end, M_mass, M_str | Non-Ran | Before-school PA | PH | Exp: Zero-hour (before-school) PA program consisting of plyometrics (short pitch, lunges, squats), stair climbing, running drills (starts, zig-zags, shuttle runs), and daily core exercises (sit-ups, leg raises).  Con: No before-school PA | Vig (50→85%HRmax​) | 12 weeks; 3 sessions per week; 45 min each | 135 | RM ANOVA | |  |  |
| 10 | Kim & Kim (56) | Ele | F&M | 18 | 18 | 3 | Cardio, Flex, M_end, M_str | Non-Ran | PE | PH | Exp: Classfit exercise as a modified type of CrossFit training in PE, which was high-intensity interval training combining aerobic, muscle strengthening, and bone-strengthening activities (e.g., duck walks, frog jumps, burpees, box jumps, and sprints)  Con: Regular PE | Vig (70−80%HRR) | 12 weeks; 3 sessions per week; 40 min each | 120 | Dep *t*-test, ind *t*-test, RM ANOVA | |  |  |
| 11 | Kim & OH (42) | High | M | 20 | 20 | 3 | Cardio, Flex, M_str | Non-Ran | After-school PA | PH | Exp: After-school circuit training using six types of park exercise equipment (cyclone running, weightlifting, cycling, sit-ups, rowing, and shoulder flexibility equipment) executed in a sequential circuit format  Con: No after-school PA program | Vig (Structural) | 8 weeks; 2 sessions per week; 9-36 min | 45 | Dep *t*-test, ind *t*-test | |  |  |
| 12 | Ko & Kim (57) | High | M | 20 | 20 | 5 | Cardio, Fat %, Flex, M_end, M_str | Ran | PE & after-school PA | Rec | Exp: Unicycling exercise in PE and after-school PA, progressed from basic balancing with a safety bar or wall, direction changes, and idling, to advanced skills like riding backward, navigating obstacles, and long-distance riding.  Con: Regular PE, no after-school PA | MVPA (Nominal) | 12 weeks; 3 sessions per week; 50–60 min each | 165 | Dep *t*-test, ind *t*-test | |  |  |
| 13 | Lee & Park (58) | Mid | F&M | 84 | 84 | 5 | Cardio, Flex, M_end, M_str | Ran | PE | PH | Exp: SPARK program applied in middle school PE, focusing on track and field (sprints, hurdles, relays), soccer (passing, dribbling, modified games), music jump rope, and fitness circuits (burpee tests, bench steps, lunges, curl-ups)  Con: Regular skill-based, teacher-directed PE | MVPA (Nominal) | 1-year program; 47 sessions in total; 45 min each | 135 | RM ANOVA | |  |  |
| 14 | Lee (59) | Ele | F&M | 52 | 50 | 4 | BMI, Cardio, Flex, M_end, M_str | Non-Ran | PE | Rec | Exp: Rhythmic jump rope in PE (3 songs) with game-based competition  Con: Regular PE | MVPA (RPE 13−15) | 10 weeks; 2 sessions per week; 50 min each | 100 | Dep *t*-test, ind *t*-test | |  |  |
| 15 | Lee (15) | Ele | F&M | 15 | 15 | 8 | BMI, Cardio, Fat %, Fat_mass, Flex, Lean_mass, M_end, M_str | Non-Ran | Sports club | Rec | Exp: Sports club Kinball program that progressed from basic ball sense and tag games to an application stage involving net-type volley games and coordination exercises, and culminating in official matches with offense/defense tactics.  Con: Regular PE | Mod (RPE 11−13) | | 12 weeks; 3 sessions per week; 50 min each | 150 | Ind *t*-test | |  |
| 16 | Moon & Lee (60) | Ele | M | 34 | 34 | 3 | Cardio, Flex, Mus_str | Non-Ran | Stu-org club | Rec | Exp: Student-organized PA club before formal classes in school. Autonomously forming groups by students, selecting sports (e.g., modified soccer, T-ball, kickball, Kinball, floorball, speed stacking, and jump rope), and adjusting their own participation rules  Con: No stu-org club | MVPA (Nominal) | 16 weeks; 3 sessions per week; 30 min each | 90 | Dep t-test, ind *t*-test | |  |  |
| 17 | Park et al.  (43) | Mid | M | 10 | 10 | 6 | BMI, Cardio, Fat %, Flex, M_end, M_str | Non-Ran | Sports club | Rec | Exp: Sport club for soccer after school, including tactical training (defense and offense tactics, step-by-step drills, small-sided games) and official league matches  Con: No sports club | Vig (60−90%HRmax​; RPE 12−17) | 12 weeks; 3 sessions per week; 45 min each | 135 | Dep t-test, ind *t*-test, RM ANOVA | |  |  |
| 18 | Park & Kim (61) | Ele | M | 10 | 10 | 4 | BMI, Cardio, Flex, M_end | Non-Ran | After-school PA | Rec | Exp: After-school Kendo program gradually progressing from learning basic martial arts skills to participating in practice sparring matches  Con: No after-school PA program | Vig (Nominal) | 16 weeks; 2 sessions per week; 100 min each | 200 | RM ANOVA | |  |  |
| 19 | Park & Kim (62) | Ele | M | 15 | 15 | 5 | BMI, Cardio, Flex, M_end, M_str | Non-Ran | Before-school PA | Rec | Exp: Zero-hour, before-school T-ball PA program structured into preparation (catch ball, base running), beginner (batting and fielding-throwing linkage), and intermediate stages (directional batting, home run challenges, and practical tactical games)  Con: No before-PA program | Mod (RPE 10−14) | 12 weeks; 2 sessions per week; 40 min each | 80 | ANCOVA, RM ANOVA | |  |  |
| 20 | Park & Moon (63) | Ele | M | 25 | 25 | 6 | Cardio, Flex, M_end | Non-Ran | Before_school_PA | Rec | Exp1: Morning soccer program selected by students (e.g., basic skill practice, formal games) Exp2: Morning PA program chosen by students (e.g., jump rope, table tennis, dodge ball, badminton) with peer interaction. Con: Reading without PA | MVPA (Structural) | 24 week; daily; 40 min | 200 | ANCOVA, Bonferroni post-hoc | |  |  |
| 21 | Sim et al.  (44) | High | M | 30 | 30 | 5 | Cardio, Fat %, Flex, M_end | Non-Ran | After-school PA | Edu | Exp: After-school soccer program league applying the Sport Education Model (e.g., league matches, modified games with participants rotating roles as referees, scorekeepers, game managers  Con: No after-school PA program | MVPA (Structural) | 12 weeks; 2 sessions per week; 40 min each | 80 | ANCOVA | |  |  |
| 22 | Yoo & Lee (64) | Ele & Mid | F&M | 96 | 93 | 3 | BMI, Flex, M_end | Ran | Sports club | Edu | Exp: Sports club PA (e.g., badminton, floorball) applying Motivational Interviewing (MI) based on the stages of behavior change, including watching health and nutrition videos, evaluating diets using a food composition bicycle, exploring exercise barriers, setting SMART goals, and completing activity logs.  Con: Sports club PA without MI | MVPA (Nominal) | | 12 weeks; 1 session per week | - | RM ANOVA | | |
| 23 | Yoon et al. (65) | Mid | F | 12 | 12 | 6 | BMI, Cardi, Fat %, Flex, M_end, M_str | Ran | After_school_PA | PH | Exp: After-school spinning cycle exercise involving upper and lower body movements synchronized with fast-rhythm music on a fixed stationary bicycle Con: After-school indoor bicycle exercise without the rhythmic upper body movements and music synchronization | Low-Mod (45−60%HRR​) | 16 weeks; 3 sessions per week; 60 min each | 180 | RM ANOVA | |  |  |
| 24 | Yoon & Park (34) | Ele | F&M | 38 | 38 | 10 | Cardio, Flex, M_end, M_str | Non-Ran | Sports club | Rec | Exp: Jump rope certification program implementing a 10-level system ranging from basic two-foot bounces (Level 10) to highly difficult single-foot double-unders (Level 1). Students advanced autonomously based on their individual skill achievement.  Con: No sports club PA | Vig (Nominal) | 10 months | - | Dep *t*-test, ind *t*-test | |  |  |

*ANCOVA*, analysis of covariance; *cardio*, cardiorespiratory endurance; *dep t-test*, dependent sample *t*-test; *edu*, educational approach; *ele*, elementary school; *F*, female; *F&M*, female and male; *flex*, flexibility; *mid*, middle school; *M*, male; *M_end*, muscular endurance; *Mod*, moderate; *M_str*, muscular strength; *n_Con*, the number of participants in the control groups; *n_ES*, the number of effect sizes reported in each study; *n_Exp*, the number of participants in the experimental groups; *non-ran*; non-randomized study; *PA*, physical activity; *PE*, physical education; *PH*, public health approach; *Rec*, recreational approach; *Ran*, randomized study; *RMe ANOVA*, repeated-measure ANOVA; *W_duration*, weekly duration, *Vig*, vigorous.

**Reference for the supplementary file**

15. Lee S. Changes in PAPS and body composition of elementary school student after participating in school Kinball sports club. *J Creat Info Cult*. (2025) 11(1):135–46.

34. Yoon YJ, Park JY. The effects of school sports club activities in elementary school students’ physical self description and changing health related components of physical fitness using jump-roping approval program. *J Res Curr Inst*. (2009) 13:133–151.

40. Hong SW, Rye JS. Effects of niche PA on the body composition and basic physical fitness of elementary school students. *Korean Assoc Learn Cent Curr Inst*. (2018) 18:461–79.

41. Kim BS, Lee CS, Oh YS. Effects of after-school PA programs on improvement of physical fitness for middle school male students. *Educ Res Inst*. (2012) 55:271–97.

42. Kim JG, Oh JS. The effects of Circuit training with outdoor exercise equipment on the health-related physical fitness and physical self-efficacy of students in a low fitness level. *J Korean Leis Sci*. (2023) 14:141–51.

43. Park JW, Koh SH, Ha SM, Kim DY. The effects of soccer club activities as part of school sports on the health-related fitness, dopamine, and cortisol of boys in middle school. *J Korean Leis Sci.* (2021) 12:21–28.

44. Sim JS, Shon JH, Yoo HS. The effect of soccer league program after school on physical fitness, physical self-concept, and academic achievement in high school students. *Korea Coach Dev Cent*. (2007) 9:115–26.

49. Hwang HJ, Kim KL, Han YS. Effects of Newsports activity during 12 weeks on health related fitness and creativity in elementary school students. *Korean Assoc Learn Cent Curr Inst.* (2015) 15:531–51.

50. Jang BW, Eom WS. Effect on PA and health of elementary school students of wearing wearable devices for 8 weeks. *J Korea Elem Educ.* (2021) 32:49–60. doi: 10.20972/kjee.32.4.202112.49

51. Jo KW. The effects of a music jump rope program on physical fitness and cognitive flexibility in elementary school students. *Korean Soc Phys Educ.* (2025) 30:357–72. doi:10.15831/JKSSPE.2025.30.5.357

52. Jung HS. Effects of increased physical activity on physical fitness and cognitive function in elementary school children. *Korean J Sport.* (2025) 23:255–60. doi:10.46669/kss.2025.23.4.020

53. Kim DH, Ban SM, Cho SC, Kuk DH. Effects of basketball training program for 12 weeks of after school on physical abilities and learning related factors in middle school students. *J Korea Acad Ind Coop Soc*. (2018) 19:186–94. doi: [10.5762/KAIS.2018.19.9.186](https://doi.org/10.5762/KAIS.2018.19.9.186)

54. Kim HS, Choi TH. A comparative study on the health related physical fitness, and school life satisfaction of elementary school students according to the participation of kids program (athletics). *J Elem Edu.* (2022) 37:151–70. doi: [10.23279/eer.37.1.202202.147](http://doi.org/10.23279/eer.37.1.202202.147)

55. Kim YY, Kang SK. Influence of zero hour class physical activities on junior high school students physical fitness, physical self-efficacy and attitude toward learning. *Korean J Meas Eval Phys Educ Spor Sci.* (2013) 15:45–56. doi: [10.21797/ksme.2013.15.1.005](http://doi.org/10.21797/ksme.2013.15.1.005)

56. Kim HC, Kim KL. The impact of Classfit training of physical fitness and prefrontal executive function in children. *Korean Assoc Learn Cent Curr Inst*. (2023) 23:745–56. doi: [10.22251/jlcci.2023.23.1.745](https://doi.org/10.22251/jlcci.2023.23.1.745)

57. Ko DJ, Kim KL. Effects of unicycle exercise on health related fitness and motor skill fitness in high school students. *J Elem Educ*. (2017) 30:1–20.

58. Lee GI, Park JY. The effect of SPARK M-SPAN based physical education curriculum (1year) on amount of MVPA and health related fitness in middle school students. *Korean J Phys Educ*. (2017) 56:713–26. doi: [10.23949/kjpe.2017.01.56.1.52](http://dx.doi.org/10.23949/kjpe.2017.01.56.1.52)

59. Lee SB. The effects of 10 weeks of music rope jumping PA on health-related physical fitness and daily stress. *J Creat Inf Cult*. (2024) 10:307–16. doi: 10.32823/jcic.10.4.202411.307

60. Moon JH, Lee DW. The effects of participation in voluntary PA club in elementary school students’ basic physical fitness and social development. *Korean J Elem Phys Educ.* (2020) 25:243–56. doi: [10.26844/ksepe.2020.25.4.243](https://doi.org/10.26844/ksepe.2020.25.4.243)

61. Park JH, Kim YH. Kumdo’s effects on body composition and physical strength of elementary school students. *Korean J Meas Eval Phys Educ Spor Sci.* (2017) 19:59–68. doi: 10.47684/jcd.2025.04.27.4.153

62. Park JS, Kim JO. Effects of 0th period T-ball physical education activities in school on health fitness and poser of elementary school students. *Korea Coach Dev Cent.* (2025) 27:153–64. doi: 10.26822/iejee.2018438134

63. Park YN, Moon JH. Effects of early morning physical activity on elementary school students’ physical fitness and sociality. *Int Electron J Elementary Educa.* (2018) 10:441–7. doi:10.26822/iejee.2018438134

64. Yoo SL, Lee S. Developing PA counseling program and verifying its effectiveness for obesity management of children and adolescents from low income family. *Korean J Sport Psychol.* (2018) 29:13–38. doi: [10.14385/KSSP.29.3.13](http://dx.doi.org/10.14385/KSSP.29.3.13)

65. Yoon JG, Kim SH, Rhyu HS. Effects of 16-week spinning and bicycle exercise on body composition, physical fitness and blood variables of middle school students. *J Excer Rehabil*. (2017) 13:400–5. doi:10.12965/jer.1735052.526
